# Evolving concepts in breast lobular neoplasia and invasive lobular carcinoma, and their impact on imaging methods

**DOI:** 10.1007/s13244-014-0324-6

**Published:** 2014-03-16

**Authors:** Tatiane M. G. Oliveira, Jorge Elias, Andrea F. Melo, Sara R. Teixeira, Salomão C. Filho, Larissa M. Gonçalves, Francesca M. Faria, Daniel G. Tiezzi, Jurandyr M. Andrade, Valdair Muglia

**Affiliations:** 1 Department of Internal Medicine (Imaging Division), School of Medicine, University of São Paulo at Ribeirao Preto, 3900 Bandeirantes Ave, Ribeirão Preto, SP Brazil; 2 Department of Gynaecology (Mastology division), School of Medicine, University of São Paulo at Ribeirao Preto, Ribeirão Preto, SP Brazil; 3 Department of Pathology, School of Medicine, Univeristy of São Paulo at Ribeirao Preto, Ribeirão Preto, SP Brazil

**Keywords:** Breast cancer, Lobular neoplasm, Invasive lobular carcinoma, Breast imaging, Resonance magnetic

## Abstract

Invasive lobular carcinoma (ILC) and lobular neoplasia (LN) are two distinct conditions that still pose challenges regarding to their classification, diagnosis and management. Although they share similar cellular characteristics, such as discohesive neoplastic cells and absence of e-cadherin staining, they represent completely different conditions. LN encompasses atypical lobular hyperplasia (ALH) and lobular carcinoma in situ (LCIS), which are currently considered risk factors and non-obligatory precursors of breast neoplasia. These lesions are diagnosed as incidental findings in percutaneous biopsies or appear as non-specific clusters of punctate calcifications in mammograms. ILC is the second most common breast malignancy and has typical histological features, such as infiltrative growth and low desmoplasia. These histological features are reflected in imaging findings and constitute the reasons for typical subtle mammographic features of ILC, as architectural distortion or focal asymmetries. Ultrasonography (US) may detect almost 75 % of the ILCs missed by mammography and represents the modality of choice for guiding biopsies. Magnetic resonance imaging (MRI) exhibits a high sensitivity for the diagnosis of ILC and for detecting synchronous lesions.

*Teaching Points*

• *LN includes ALH and LCIS, risk factors and non-obligatory precursors of breast cancer*.

• *Absence of e-cadherin staining is crucial for differentiation among ductal and lobular lesions.*

• *ILC has typical histological features, such as infiltrative growth and low desmoplasia.*

• *Mammographic features of ILC are often subtle and reflect the histological features.*

• *MRI exhibits a high sensitivity for the diagnosis of ILC and for detecting synchronous lesions.*

## Introduction

Although invasive lobular carcinoma (ILC) and lobular neoplasia (LN) have been described and recognised for many years, their classifications continue to be controversial, creating diagnostic problems and debates concerning the most appropriate treatment and follow-up for these patients [1, 2].

The role and implications of LN in the physiopathology and development of breast cancer are not fully understood [3]. In addition, the majority of cases have no clinical signs or imaging features, making this a challenging diagnosis. With the increasing number of core needle biopsies, diagnosis of LN has become more frequent, increasing the necessity for improvements in the knowledge of the different aspects influencing the decision toward surgical excision or conservative management [1, 2].

ILC represents approximately 5–15 % of all breast carcinomas and exhibits challenging characteristics, such as low sensitivity in screening examinations, tumour size underestimation in both mammograms and clinical examinations, and a high prevalence of synchronous lesions [4]. Knowledge of the main imaging findings, as well as of the imaging limitations, may contribute to an early diagnosis and more effective therapies.

This article reviews the concepts of ILC and LN and their main histological variants. Their clinical, histopathological, and imaging features are indicated emphasising the key pathological and radiological findings for most accurate patient therapeutic decisions and follow-up.

## Lobular neoplasia

### Background

Lobular carcinoma in situ (LCIS) was first documented by Ewing in 1919 and was described by Foote and Stewart in 1941 [5].

In 1978, Haagensen proposed the term “lobular neoplasia” to group two histologically similar lobular proliferations: atypical lobular hyperplasia (ALH) and LCIS. In fact, rather than including two proliferative diseases, LN encompasses a continuum between these two lesions [1, 4].

**Fig. 1:**
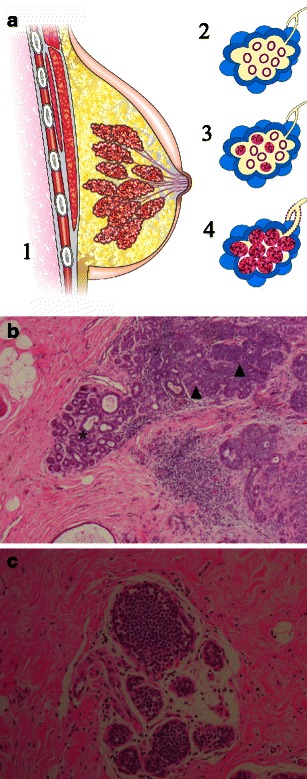
**a** Schematic drawing: *1* sagittal view of the breast showing terminal duct-lobular units converging to lactiferous ducts and papilla; *2* a normal terminal duct-lobular unit (TDLU); *3* atypical lobular hyperplasia (ALH), cellular proliferation in less than 50 % of acini; *4* lobular carcinoma in situ (LCIS) cellular proliferation expanding more than a half of acini within a TDLU. (**b**) ALH in histological slice (haematoxylin–eosin [H-E] stain, ×40 photomicrograph) demonstrating some normal acini (*) and some lobules distended by cell proliferation (*black arrowheads*). **c** LCIS in histological slice H-E, ×200, showing all acini filled and distended by an uniform cell population

The distinction between ALH and LCIS is based on the degree of acini involvement. According to Page et al., LCIS should be used to define cellular proliferations involving and distending more than half of the lobular unit. ALH is used to define proliferations when the criteria for LICS are not met and when less than half of a lobular unit is involved (Fig. 1) [4].

In the 1970s, Haagensen, Rosen and Page described LCIS as a risk factor for breast cancer, emphasising its indolent behaviour and its interval of 15–20 years before becoming an invasive lesion. However, they had already noticed that LCIS lead to invasive progression less frequently than high-grade ductal carcinoma in situ (DCIS), confirming a distinct biological behaviour [1].

Currently, it is estimated that ALH increases the risk for breast cancer approximately fourfold to fivefold, whereas LCIS is accompanied by a ninefold to tenfold increase [3]. The risk is higher in women with breast carcinoma family history [2].

The concept that LN does not constitute a true pre-neoplastic lesion, but rather represents a risk factor, was supported by evidence showing that LN increases the risk of cancer in both breasts, the most common histological type of which is invasive ductal carcinoma (IDC) [1, 3].

However, new evidence from molecular and genetic analyses also points to a precursor role for LN based on the following: (1) the 3-times-greater risk of developing breast cancer in the ipsilateral, compared to the contralateral breast; (2) the histological and molecular similarities between LN and ILC; and 3) the higher percentage of patients with a history of LN who were found to develop ILC versus IDC compared with the general population of women with breast cancer [1, 3].

Therefore, LN has been defined as an entity that includes both ALH and LCIS and qualifies as a non-obligate precursor lesion, as well as a risk indicator for breast carcinoma [1, 3, 4, 6, 7].

The latest World Health Organisation classification of tumour groups adopts the term LN without consideration of its subtypes, ALH and LCIS [8].

### Clinical features

The peak incidence of LN occurs at approximately 40–50 years of age, and LN has a clear predominance in premenopausal women [1, 3]. Typically, multifocal and bilateral lesions occur in up to 50 % and 30 % of cases, respectively [1, 3, 4].

The true incidence of LN is not known due to the associated asymptomatic conditions and paucity of imaging findings. LN usually does not have a characteristic imaging finding, and most of the diagnoses are made based on incidental features in excisional biopsies. Some studies have described LN as an incidental finding in approximately 0.5–3.8 % of benign breast lesion biopsies [1, 3].

### Histopathology

The classic form of LCIS is diagnosed by the following typical cellular patterns of small, uniform, and loosely cohesive cells, with small nuclei, few pleomorphism, high nuclear-to-cytoplasmic ratio and clear cytoplasm vacuoles known as magenta bodies.

These cells fill acini in a discohesive pattern, respecting the lobular architecture and they can extend along ducts infiltrating between the intact myoepithelial membrane and the ductal epithelium in a classic arrangement known as the Pagetoid spread [1, 4].

The immunochemistry study shows hormonal oestrogen and progesterone receptors positive in 60–90 % of cases, whereas the *HER2* is usually not overexpressed [1].

**Fig. 2:**
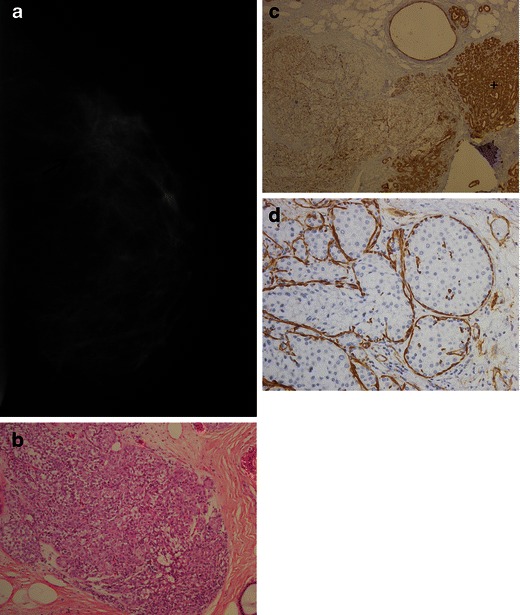
A 47-year-old woman; percutaneous sample shows LN (LCIS and ALH). The final diagnosis was confirmed by surgical biopsy. **a** Mammography in cranio-caudal view reveals a focal asymmetry with subtle architectural distortion in the lateral quadrant. **b** Histological H-E stain slice (×100) with marked lobular distention and discohesive cells. **c** Immunohistochemical (IHC)—photomicrography staining (×40) for e-cadherin showing no expression in acini involved by LN, on the left and strong expression on preserved acini, on the right (+). **d** IHC for smooth muscle actin (×200), a myoepithelial membrane marker. There is strong reactivity for the cytoplasm of basal membrane, confirming an intact layer of epithelial cells without invasion. Notice the marked loss of cell cohesion, without forming papillary or cribriform arrangement like in some of DCIS

Another important phenotypic characteristic is the lack of E-cadherin expression in LN. E-cadherin is a transmembrane glycoprotein involved in cell adhesion. This glycoprotein is found on normal breast tissues and is strongly expressed in ductal neoplasm [9]. Thus E-cadherin immunostaining is a tool extremely useful for the differentiation of ductal and lobular carcinomas (sensitivity of 94 % and specificity of 98 %) (Fig. 2).

Other markers of LN include the cytoplasmic localisation of p120-catenin and cytokeratin-34betaE12 [2].

Despite lacking a genetic signature, losses of chromosomal material on 16q and gains on 1q were detected in LN, similarly to columnar cell lesions (CCLs), low-grade DCIS, tubular carcinoma (TC), and ILC. This result suggests a common evolutionary pathway for low-grade invasive and in situ lesions [1].

### Imaging findings

**Fig. 3:**
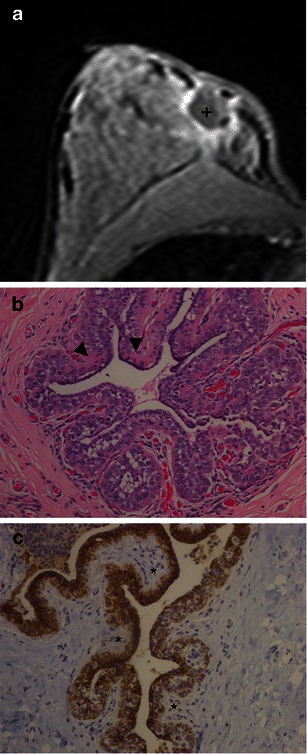
A 40-year-old woman with previous surgical resection of an ILC 15 months ago. Mammogram (not shown) with architectural distortion, possibly surgical-related change. **a** MR image axial T1, 3rd min, post-contrast media. There is a collection with low signal (+) and marked wash-in enhancement surrounding the surgical area. A new surgical exploration showed LN. **b** Photomicrograph H-E stain, ×200, pagetoid ductal spread, with lobular proliferation, extending to the duct, between the basal membrane and epithelial cell (*arrowhead*). In **c** the epithelial cells show marked reactivity expression for e-cadherin and e-cadherin-negative LCIS cells (*)

Rendi et al. [10] studied 93 cases of LN and described that, upon examination of the imaging findings, 74 %, 24 %, and 2 % of cases were detected by mammograms, magnetic resonance imaging (MRI) and US, respectively. Microcalcifications were the most common finding, occurring in 69 % of cases, followed by pathological MRI non-mass enhancement in 16 % (Fig. 3), masses in 14 %, and architectural distortions in 1 % of cases.

**Fig. 4:**
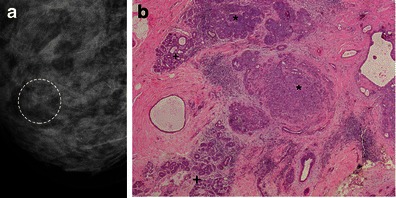
A 43-year-old woman: routine mammography for screening. **a** Additional cranio-caudal magnified views showing new cluster of microcalcifications. The patient underwent a percutaneous biopsy and, after that, surgical excision of the residual microcalcifications. **b** Histology confirms a LN, with less than 50 % of acini involved. Normal lobular acini (+) and ALH (*) are easily seen

Mammography is the most sensitive method for diagnosing LN. The most typical finding is clusters of punctate microcalcifications, seen in 30-50 % of cases (Fig. 4). In fact, these clusters often are imaging findings of associated lesions such as sclerosing adenosis, columnar cell hyperplasia, and spherulosis [2, 4, 10] adjacent to LN. However, it is important to emphasise that majority of cases of LN have no imaging findings and represents incidental findings in histopathological specimens.

### Histological variants

New molecular techniques combined with immunohistochemical (IHC) markers have enabled the characterisation of histological variants of LCIS. Pleomorphic lobular carcinoma in situ (PLCIS) is an example of a new entity with a more aggressive biological behaviour that may exhibit apocrine or histiocytic differentiation and signet ring cells.

PLCIS typically has marked pleomorphism, and comprises nuclei that are 2–4 times larger and more discohesive cell pattern than those of classic form of LCIS. Necrosis and calcifications are more frequent and may mimic DCIS, particularly the low-grade, solid DCIS [1, 2].

In contrast to its counterpart LCIS, PCLIS occurs predominantly in postmenopausal women; in the majority of cases patients present with clusters of microcalcifications on their mammograms [6].

Immunohistochemistry is essential for the diagnosis of the pleomorphic variants. These variants are characterised by a combination of an absence of E-cadherin expression, a lower expression of oestrogen and progesterone receptors, a higher expression of *HER2*, a higher *Ki67* index, and a higher positivity for *cytokeratin GCDFP-15*, compared with classic forms.

Currently, the finding of PLCIS in a percutaneous biopsy indicates surgical excision with free margins [2]. However, the most important feature of PLCIS is its association with ILC, which is found in approximately 50 % of cases [6, 11].

### Clinical management and recommendations

When a diagnosis of LN is made based on a core needle biopsy (CNB), the risk of diagnosis underestimation is approximately 25 % [12, 13]. Even with satisfactory sampling and radiological-pathological concordance, this risk can be up to 9 % [14].

According to Middleton et al., the presence of a mass or architectural distortions were the main findings associated with the diagnostic underestimation of LN in percutaneous breast biopsies [15].

Others studies have suggested that when histological samples with four or more ductulo-lobular units involved in LCIS are present, there is a higher risk for underestimating the diagnosis, as well as a higher association with invasive carcinoma [11].

However, after diagnosis of LN, there is no consensus on further management yet. Prospective studies are required to define which patients really could benefit from surgical excision [3, 10, 11].

Surgical excision must be performed in cases in which there is a presence of other risk factors for breast cancer, pleomorphic lobular carcinoma in situ, equivocal histopathological or immunohistochemical findings preventing the differentiation between LN and DCIS, or radiological-pathological discordance [1, 3, 11]. The main reasons for radiological-pathological discordance are the occurrence of a lesion presenting as a mass or distortion, microcalcifications that were not fully included in the biopsy specimen and insufficient material for histopathological diagnosis [1, 3].

However the majority of authors, based on the upgrade rate to malignancy, support that surgical excision should be done in all LN diagnosis [11, 14].

Nevertheless, it is important to consider that when a diagnosis of LN is made after an excisional biopsy or when it is an incidental finding of an oncologic surgery, no free surgical margins are required, except in cases of a pleomorphic subtype of LN.

The use of radiation therapy and endocrine chemoprophylaxis are controversial and there is no consensus on their use and efficacy [3].

A proposed follow-up for patients with a prior diagnosis of LN is a clinical examination every 6 months and an annual mammogram starting from the date of diagnosis. For women with no other associated risk factors, there is no formal indication for an MRI screening [3].

## Invasive lobular carcinoma

### Background

In 1941, Foote and Stewart [5] described LCIS and found similarities between their cytological patterns and those of ILC, highlighting the common origin of the terminal duct-lobular unit for both conditions. When diagnosis is based strictly on the criteria of Foote and Stewart, invasive lobular carcinoma usually constitutes 5 % or less of breast carcinomas [1].

However, currently, less strict criteria are employed, and ILC corresponds to approximately 5–15 % of all breast carcinomas, representing the second most common type of breast malignancy [4].

The classic form of ILC is the most common subtype and may coexist with LN in up to 90 % of cases [16]. Other subtypes, including the alveolar, solid, pleomorphic and tubulolobular subtypes, have similar cellular features but different structural arrangements, molecular aspects and clinical behaviours.

### Clinical features

The peak incidence of ILC occurs in post-menopausal women in their 50s and 60s, with a mean age that is approximately 1–3 years older than the mean age of women with invasive ductal carcinoma [17].

A common clinical presentation is a palpable mass or thickening of the breast tissue, often with imprecise delimitation and size underestimation at clinical examination.

A marked characteristic of ILC is the presence of synchronous neoplastic foci, occurring in the same or in the contralateral breast (multifocality/multicentricity), which happens more frequently than in IDC. The relative risk to bilaterality is 1.5 compared with invasive ductal carcinoma [17].

The pattern of haematogenic metastases associated with ILC shows some peculiarities. There is a higher rate of metastasis in bones, gastrointestinal tract, uterus, meninges, ovaries and serosas, as well as a lower rate of lung metastases, in ILC compared with IDC [18].

### Histopathology

The histology of ILC is similar to that of LN; specifically, the cells are small, discohesive and uniform, with a small cytoplasm, cytoplasmic Magenta Bodies and a low mitotic index [2].

**Fig. 5:**
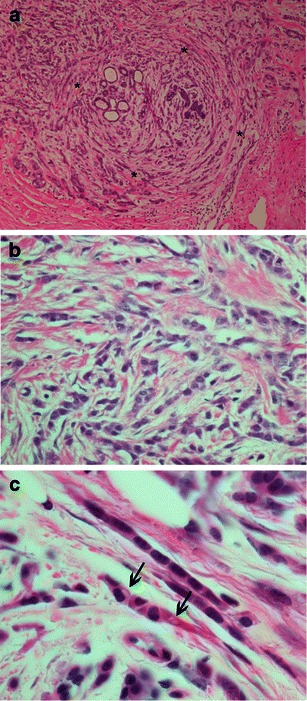
ILC classic type, H-E photomicrographs. **a** Magnification ×40. Small cells infiltrating breast stroma surrounding benign breast tissues in a targeted manner (*). **b** Magnification ×200, showing cells arranged in lines, with interspersed fibrosis and inflammation. **c** Magnification ×1,000, in oil immersion, cells lying in an “Indian file” manner and with cytoplasmactic vacuoles, Magenta Bodies (*arrows*)

Neoplastic cell proliferation occurs, preserving the breast parenchymal architecture and associated to a low level of host desmoplastic reactions. The histological diagnosis is based on two classic patterns of the arrangement of neoplastic cells in the glandular stroma: (1) single-file linear cords dispersed throughout a fibrous tissue (so-called Indian file) and (2) concentric cell arrangements involving ducts and lobules, described as a “targetoid” appearance (Fig. 5).

Although it was initially questioned, the current grading of ILC is performed according to the Nottingham classification, which demonstrates a correlation between grading, survival and disease-free intervals [2]. Approximately 76 % of lesions are grade II; usually, most of the grade III lesions arise from variants of ILC (non-classic subtypes). The mitotic index is the single most important predictor of prognosis [2].

Most (90 %) of the lesions have hormonal receptors, but HER2 overexpression is rarely seen in ILC [19, 22].

The absence or discrete expression of E-cadherin is the major IHC marker of the differentiation between ILC and invasive ductal carcinoma. Occasionally, the extension of an IDC to the interior of a lobule, called “lobular cancerisation” may mimic an ILC. In this situation, E-cadherin staining is crucial for obtaining the correct diagnosis.

Although there is no specific genetic profile for ILC, one of the main genomic changes is the loss of material in the short arm of chromosome 16, the location of the gene for E-cadherin. The E-cadherin gene has been postulated as a possible tumour gene suppressor with a large number of possible mutations and is also found in ILC and low-grade DCIS [20]. This finding suggests a potential role for E-cadherin in the pathogenesis of breast cancer [23]. Other IHC markers that may be used for ILC characterisation include cathepsin D, cyclin D1 and Bcl-2 intracytoplasmic localisation of p120-catenin [2, 5].

### Imaging findings

#### Mammography

Typically, ILC has low mammographic sensitivity, varying from 57 to 79 %, and is one of the most important causes of false-negative mammograms, with rates up to 20 % [16, 21]. The histological features of ILC, such as a lack of stromal desmoplasia and an infiltrative growth pattern, account for the low sensitivity of mammography due to the associated subtle findings and underestimation of the size of lesions [21–23]. Lesions as large as 5.0 cm may be missed in mammography if they exhibit similar density to breast parenchyma.

**Fig. 6:**
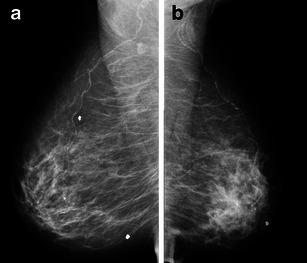
A 56-year-old woman with ILC. Mammography in oblique views showing global asymmetry with increased parenchymal density, architectural distortion and loss of volume in the left side

**Fig. 7:**
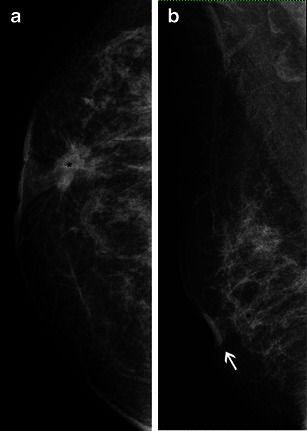
A 42-year-old woman with ILC. **a** and **b** Cranio-caudal and oblique views showing an irregular mass, with spiculated margins and isodense to parenchyma (*). There is a nipple retraction (*arrow*). The mass is much more conspicuous in cranio-caudal view

Subtle presentation, including focal asymmetry and architectural distortions, is more common in ILC than in IDC [24]. In larger lesions, a classical presentation of increased parenchymal density and a reduction of breast volume may be found (Fig. 6) [16]. However, the most common finding of ILC is an irregular mass, with indistinct or spiculated margins (Fig. 7). Calcifications are less frequent in ILC, occurring in about 10-20 % of cases [25].

#### Ultrasound

Ultrasonography plays an essential role in the evaluation of ILC, with sensitivity ranging from 81 to 83 % [26]. It exhibits a higher accuracy than mammography for lesion measurements, detections of multifocality and multicentricity and lymph node assessments [16]. It is the primary method for guiding biopsies of index lesions and adenopathy [26, 27], as well as for the assessment of palpable lesions not seen on mammograms. Up to 73 % of carcinomas not seen on mammograms may be detected through this method [26].

**Fig. 8:**
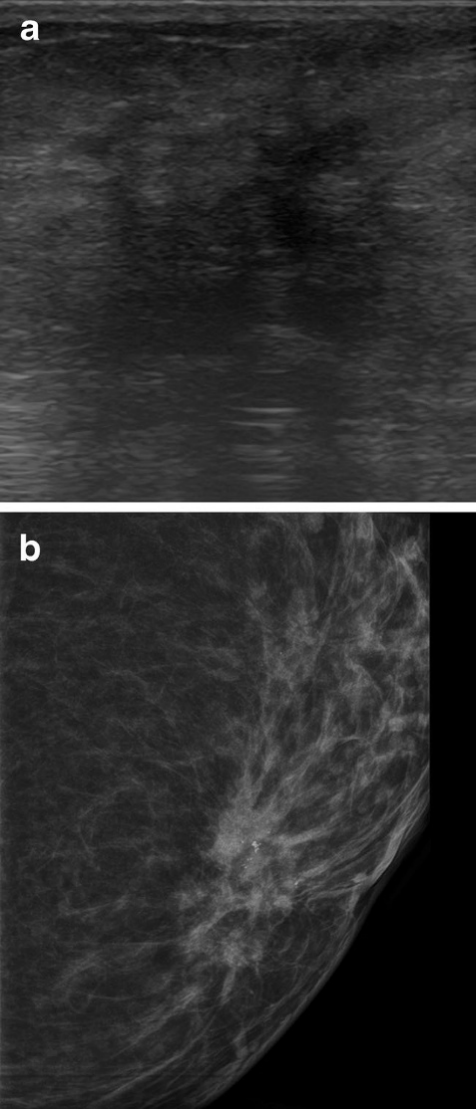
A 73-year-old woman with ILC. **a** US image depicts an irregular, hypoechoic mass, with indistinct margins and distal attenuation of sound. **b** Magnified cranio-caudal view also shows an irregular mass associated to microcalcifications

The sonographic findings of ILC may be similar to those of IDC. The most common finding is a hypoechoic, irregular mass with indistinct margins in approximately 85 % of cases (Fig. 8) [28]. For the detection of small lesions (smaller than 1 cm), the use of high-frequency transducers coupled with harmonics may enhance the detection of microlobulations and spiculations, thereby improving diagnostic accuracy [25].

A typical finding of ILC is the lower frequency of “taller than wider” lesions compared with those found in IDC. Cawson et al. [29] attributed the preferential parallel orientation of ILC in the US images to the infiltrative growth pattern that follows the anatomical breast parenchymal planes.

Although atypical for a malignant mass, hyperechoic lesions are 10 times more common in ILC than in other types of breast malignancies [29]. The posterior acoustic shadow without a definite mass may be a presentation of ILC [28].

#### Magnetic resonance imaging

**Fig. 9:**
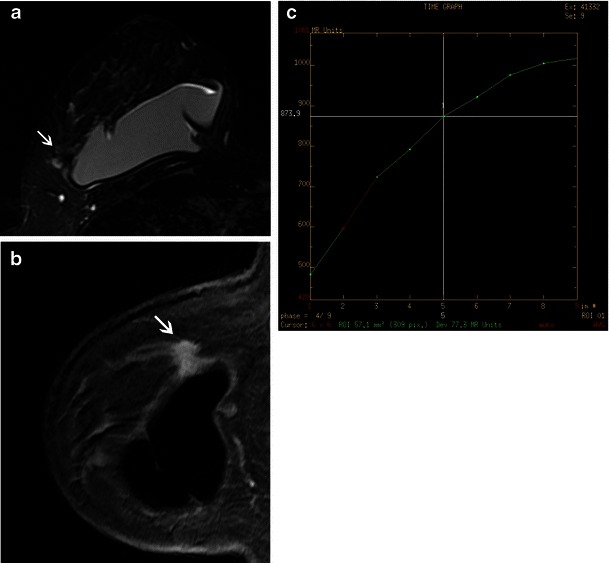
A 45-year-old woman with ILC. **a** and **b** MR images from right breast, respectively, T2 with fat suppression, sagittal reformation from T1 volumetric, post-contrast images, showing an irregular mass (*arrows*), with spiculated margins, seen adjacent to an intact breast implant. After IV contrast, there is a uniform, progressive enhancement, until delayed phases, typical of invasive lobular neoplasia. (**c**) Kinetic curve type 1

**Fig. 10:**
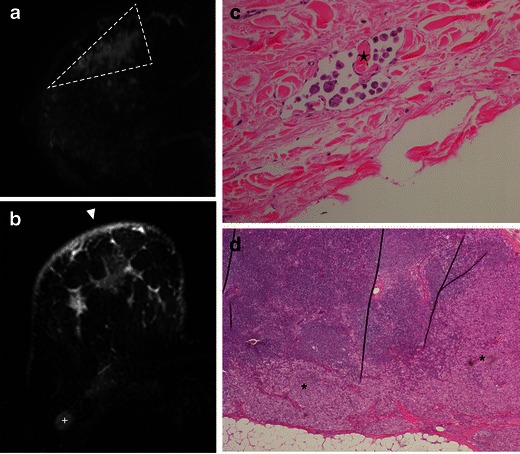
A 56-year-old woman. **a** MR T1-post-contrast subtracted sagittal image showing a non-mass enhancement, with segmental distribution, in an extension larger than seen on mammogram view (*dashed line*). **b**) MR—axial STIR demonstrates cutaneous oedema (*arrowhead*) and axilar adenopathy (+). **c** H-E ×200, showing tumour embolisation in a dermal lymphatic (*star*). **d** Sentinel lymph node biopsy with cortical invasion (*), in H-E stain, ×40

MRI shows great sensitivity for ILC, ranging from 83 to 100 % [16]. The most common MRI finding associated with ILC is an irregular and spiculated mass-like lesion (Fig. 9), followed by a non-mass lesion in 20–40 % of cases (Fig. 10) [30].

The kinetics of ILC in dynamic post-contrast images show a slow-rising signal intensity, characteristic of a type I or progressive curve, which is the most common finding in kinetics analysis. Peripheral oedema and the rim pattern of enhancement are less common findings [30].

Compared with mammography and US, MRI is the best option for determining the size of lesions, although overestimation occurs in up to 20 % of cases. Overestimation has been associated mainly with the presence of LCIS. Despite being less common, diagnostic underestimations may occur and are frequently associated with larger lesions and higher pathological grades.

**Fig. 11:**
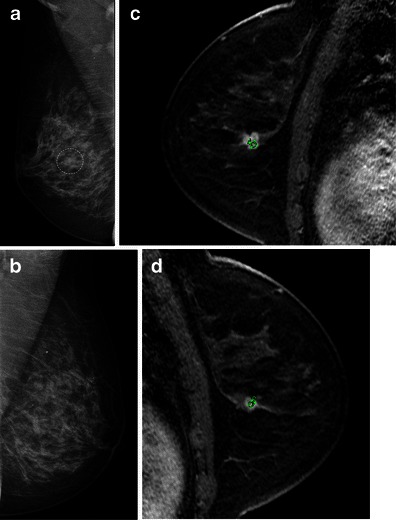
A 49-year-old woman with bilateral ILC. **a** and **b** Mammographic oblique views for screening. A dense breast is seen, with a small irregular lesion, indistinct margins in the central right breast (*dotted line*). Core needle biopsy confirmed an ILC and MR exam was requested. Sagittal views, T1 post-contrast, from right (**c**) and left breasts (**d**) confirming the right lesion seen on mammography (larger in MR images). However, another lesion is seen in contralateral breast, also with irregular margins and strong enhancement (*circle*). The kinetic curves for index lesion and synchronous contralateral mass were type 3 and 2 curves, respectively, not showed

In addition, MRI shows a high accuracy for the detection of synchronous lesions in the same or contralateral breast, ranging from 80 to 90 % (Fig. 11) [30].

A more precise determination of the burden of lesions allows for better surgical planning (mastectomy versus conservative techniques) and is fundamental for the indication and planning of neoadjuvant chemotherapy [31].

Despite the evidence of the high sensitivity of MRI for the detection of ILC, its role in preoperative staging is still controversial. In fact, no study has confirmed an improvement in overall survival. A possible explanation for this result is that MRI shows small synchronous lesions that would be effectively treated by adjuvant therapies (chemotherapy and/or radiotherapy) without the need for additional surgical procedures. However, most of the synchronous lesions diagnosed by MRI share histological characteristics related to aggressiveness and a prognosis similar to that of the index lesions [30]. Furthermore, the impact of a lesion’s overestimation by MRI when deciding and planning the surgical approach is questionable, as this could arguably lead to overtreatment [31].

Current evidence has shown changes in the clinical management of patients who had a preoperative breast MRI [17] and the clear benefit of a minor rate of surgical re-assessment in patients who underwent conservative surgeries. Mann et al. [32] retrospectively evaluated the role of preoperative MRI in the treatment of ILC and concluded that MRIs significantly reduced the rate of re-assessment in breast conservation surgeries, without increasing the number of mastectomies.

### Prognostic factors and clinical management

Although ILC has some good prognostic factors, such as lower grades, lower mitotic indexes, positive hormonal receptors and a lack of HER2 overexpression, there is no difference in the survival rates of patients with these two histological types [26], which may be justified by the fact that, usually, ILC is diagnosed in more advanced staging than IDC [33].

It is well known that when conservative procedures are chosen, free surgical margins are less common for ILC than for IDC; thus, the rate of re-excision is significantly higher for ILC treated with conservative surgeries [34]. Mastectomy as an option for re-assessment is used in 16–48 % of patients [30]. However, when surgical margins are free in conservative surgeries, there is no difference in local recurrence, disease-free survival or overall survival compared with IDC [26]. Accordingly, the indications for surgical treatment, radiotherapy and chemotherapy do not differ between ILC and IDC [2]. Endocrine therapy is often used in women with ILC, given their high proportion of hormonal receptor expression [2].

### Histological variants

The histological variants of ILC exhibit the typical cytological features of lobular carcinoma in the classic form: uniform neoplastic cells that have round or notched ovoid nuclei, thin-rimmed cytoplasms with an occasional intracytoplasmic lumen and immunohistochemically negative E-cadherin. The main differences between the variants arise from their growth patterns and structural arrangements.

**Fig. 12:**
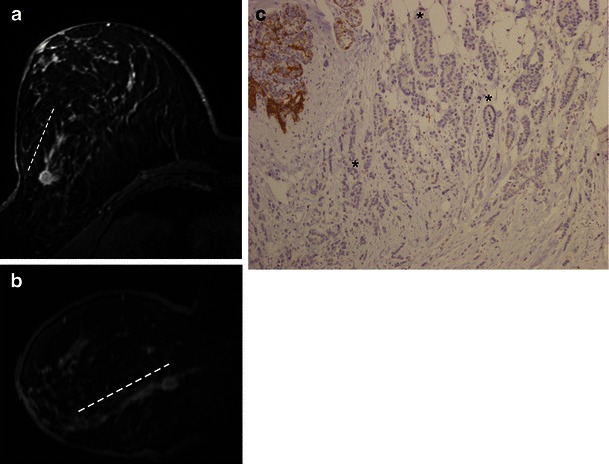
Tubulo-lobular variant in a 59-year-old woman with non-palpable lesion. **a** and **b** MR images, axial STIR and MIP reconstruction from T1 post-gadolinium, showing an irregular mass, spiculated margins in the transition of outer quadrants of right breast. There is a clear ductal extension (trace) towards the nipple. **c** Histological slice, e-cadherin stain, ×100, demonstrating neoplastic cells in the classic pattern of slender strands interposed to tubular structures (*) with no expression for e-cadherin

The main variants include the alveolar, solid, tubulolobular (Fig. 12) and pleomorphic forms. Their main characteristics are summarised in Table 1. Based on the imaging findings, the differentiation of various subtypes of ILC is not possible. This diagnosis is made solely through histology, and its prognosis is variable [2, 5].

**Table 1:** Histological variants of invasive lobular carcinoma

Subtype	Histological features
Alveolar	Small cells arranged in more-or-less globular aggregate of 20 or more cells separated by thin bands of fibrous stroma.
Tubulo-lobular	Neoplastic cells form tubular, glandular structures. The best prognosis among all forms.
Solid	Solid sheets of uniform small cells showing lobular morphology.
	There are more pleomorphism and higher frequency of mitoses.
Pleomorphic	The more aggressive and worst prognosis.
	The histological pattern may mimic an undifferentiated ductal carcinoma. Cells are larger due to irregular nuclei with prominent nucleoli.
	Mitotic figures are frequent, disperse or in line, throughout the breast stroma. Apocrine or histiocytoid differentiation is common.

**Fig. 13:**
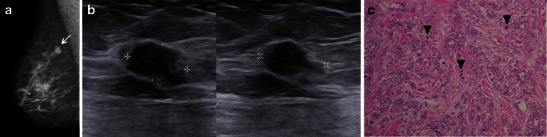
A 61-year-old woman with PILC. **a** Oblique view showing an irregular, hyperdense mass in axillar extension (*arrow*). **b** US images depict an irregular, hypoechoic, parallel lesion, with angulated margins (calipers). **c** Photomicrograph H-E stain, ×200, the intense pleomorphism is evident, along with large nuclei and heterogeneous chromatin (*arrowhead*)

Pleomorphic invasive lobular carcinoma (PILC), although rare, is the most clinically important variant due to its aggressive biological behaviour. Although it shares genetics and IMC features with the classical form of ILC, including its positivity for hormonal receptors and its lack of E-cadherin expression, the diagnosis of PILC is related to an overexpression of HER2, a more advanced clinical staging, a higher incidence of multicentricity, a higher early recurrence and a slightly higher overall recurrence [35]. The radiological findings of PILC are similar to those of classic ILC, and a mass with spiculated or indistinct margins is the most prevalent feature, followed by architectural distortions (Fig. 13) [35].

**Fig. 14:**
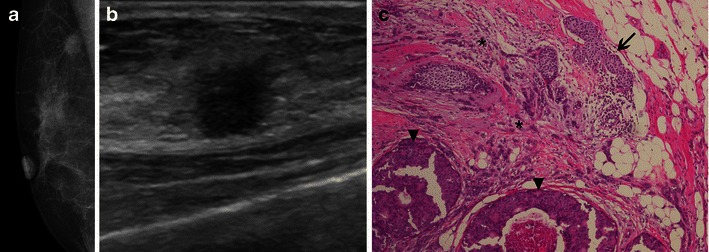
A 61-year-old woman with right breast lump, diagnosed as mixed carcinoma, ducto-lobular. **a** Oblique mammography view showing an irregular, spiculated mass. **b** US showing an irregular, non-parallel mass, surrounded by an echogenic halo. **c** H-E stain ×100, LCIS is seen in the right upper corner (*arrow*), ILC in the centre (*) and DCIS (*arrowheads*) at the bottom of the figure (IDC not present in the same slice)

Tumours with a mixed ductal and lobular features account for 2–6 % of breast carcinomas, exhibiting features that are more related to ILC and a prognosis that is more dependent on phenotypic characteristics, which are generally better than those of pure IDC (Fig. 14) [18].

## Conclusion

LN includes AHL and LCIS, and is a non-obligate precursor lesion and risk factor for breast cancer. The findings of LN in percutaneous biopsies may be underestimated in up to 24 % of cases, prompting excisional biopsies when there is a radiological-pathological discordance or the presence of other risk factors or PLCIS variant.

ILC is the second most common histological type of breast cancer. Mammography has a lower sensitivity for ILC than it does for IDC, and the typical subtle findings may lead to false-negative exams. MRI is more accurate for determining lesion sizes and detecting synchronic lesions. The histological variants of ILC are indistinguishable from the classic type by imaging alone, even for the pleomorphic variant, which is the most aggressive ILC variant.

In conclusion, LN and ILC are two distinct conditions that share similar cellular characteristics, but with different prognosis. It is important for radiologists be familiar with evolving pathological concepts of LN and ILC to improve diagnostic accuracy and confidently recommend clinical follow-up or surgical management.
